# Short-Term Peg-IFN α-2b Re-Treatment Induced a High Functional Cure Rate in Patients with HBsAg Recurrence after Stopping Peg-IFN α-Based Regimens

**DOI:** 10.3390/jcm12010361

**Published:** 2023-01-02

**Authors:** Fengping Wu, Yikai Wang, Dandan Cui, Yan Tian, Rui Lu, Chenrui Liu, Mei Li, Yaping Li, Ning Gao, Zicheng Jiang, Xuemei Li, Song Zhai, Xin Zhang, Xiaoli Jia, Shuangsuo Dang

**Affiliations:** 1 Department of Infectious Diseases, the Second Affiliated Hospital of Xi’an Jiaotong University, Xi’an 710004, China; 2 Department of Infectious Diseases, Ankang Central Hospital, Ankang 725000, China

**Keywords:** HBsAg recurrence, peginterferon alpha, re-treatment, HBsAg clearance, HBV vaccine

## Abstract

Little is known about the treatment of patients with hepatitis B surface antigen (HBsAg) recurrence after being clinically cured by peginterferon alpha(peg-IFN-α)-based regimens. This study aimed to investigate the efficacy and safety of peg-IFNα-2b in re-treating patients with HBsAg recurrence after stopping peg-IFN α-based regimens. In this two-center, prospective observational study, 33 patients with HBsAg recurrence after stopping peg-IFN α-based regimens were enrolled and re-treated with an individualized course of peg-IFN α-2b. The hepatitis B virus (HBV) vaccine could be injected immediately after HBsAg clearance, according to patients’ willingness. All patients were monitored and followed-up for 48 weeks after peg-IFN α-2b re-treatment stop. The primary endpoint was HBsAg clearance at the end of follow-up. At baseline, all patients had HBsAg levels of <10 IU/mL and undetectable HBV DNA, with the median HBsAg level of 1.66 (0.56–2.87) IU/mL. After a median of 24 (24–30) weeks of peg-IFN α-2b re-treatment, 87.9% (29/33) of the patients achieved HBsAg clearance again and 66.7% (22/33) of the patients achieved HBsAg seroconversion. At the end of follow-up, the HBsAg clearance and HBsAg seroconversion rates decreased to 78.8% (26/33) and 51.5% (17/33), respectively. Furthermore, 88.9% (16/18) of the patients with HBsAg clearance benefited from receiving the HBV vaccine therapy. Generally, both peg-IFN α-2b and HBV vaccine therapy were well tolerated. A high functional cure rate can be achieved by a short-course of peg-IFN α-2b re-treatment in patients with HBsAg recurrence after stopping peg-IFN α-based regimens. Furthermore, injecting HBV vaccine is beneficial after HBsAg clearance.

## 1. Introduction

Chronic hepatitis B virus (HBV) infection remains a major global health problem. Hepatitis B surface antigen (HBsAg) clearance, which is currently regarded as the functional cure of chronic hepatitis B (CHB), is associated with improved long-term outcomes and reduced risk of complications, and is therefore considered to be the ideal goal of anti-HBV therapy [1].

However, the HBsAg clearance rate is relatively low (<12.0%) when using the existing antiviral drugs monotherapy [2,3,4,5]. Over the past few decades, many efforts have been made to optimize therapeutic regimens to improve HBsAg clearance and have obtained promising results. Our recent studies, as well as those of others, suggested that using combination regimens with nucleos(t)ide analogues (NAs) and peginterferon alpha (peg-IFN-α) to treat CHB patients with stably suppressed HBV DNA and low HBsAg levels could improve the HBsAg clearance rates of up to 20.0%–66.67% [6,7]. Furthermore, high HBsAg clearance rates of up to 44.7%–47.9% could be achieved by 48–96 weeks of peg-IFNα-based therapy in inactive HBV carriers (IHCs) [8,9]. 

Unfortunately, the status of functional cure is not persistent, due to the fact that it just represents a status of immunological control of HBV infection, rather than curing HBV infection completely, that is, the true eradication of HBV covalently closed circular DNA [10]. Therefore, individuals with the functional cure are still at risk of HBV reactivation. A study from Korea that reviewed 110 patients who achieved functional cure by NAs therapy showed that 7% of the patients experienced HBsAg recurrence after treatment discontinuation [11]. A retrospective study from the United States demonstrated that the 5-year cumulative risk of HBsAg seroreversion was 5.4% in patients with spontaneous and treatment-related functional cure [12]. An observational study from China enrolled 238 cases with functional cure induced by IFN-α-based regimens and showed that the cumulative rates of HBsAg recurrence were 6.29%, 8.18%, and 9.66% for 52 weeks, 104 weeks and 597 weeks after treatment cessation, respectively [13]. In addition, another prospective study from China observed 176 HBeAg-negative CHB patients with functional cure by standard INF-α or peg-IFN α-2a therapy and showed that the cumulative rates of HBsAg recurrence and HBV DNA recurrence were 12.79% and 2.33%, respectively, during 48 weeks of follow-up after treatment cessation [14].

Recurrence of HBsAg or HBV DNA after functional cure not only places great physical and mental burdens on patients but also brings new barriers and challenges to the functional cure of chronic HBV infection. How to achieve functional cure again by using the existing antiviral drugs remains an interesting problem to solve and has aroused great attention in researchers and clinicians. Our center and the Ankang Central Hospital have made a long-term commitment to investigate the functional cure of chronic HBV infection by peg-IFN α-based regimens. A total of 271 patients have achieved functional cure by peg-IFN α-based regimens in the past 7 years, including 48 patients treated with peg-IFN α-2a and 223 patients treated with peg-IFN α-2b. During a median of 110 weeks of follow-up, 35 patients experienced HBsAg recurrence (Rates of HbsAg recurrence for peg-IFN α-2a and peg-IFN α-2b treatment were 14.6% (7/48) and 12.6% (28/223), respectively). Most of them were willing to receive peg-IFN-α re-treatment to pursue functional cure again.

The Chinese-made peg-IFN α-2b (PegBeron^®^, Y shape, 40 kD) was marketed in China in 2016, which is more stable in structure and is smaller in immunogenicity than Peg-IFN α-2a (Pegasys^®^, U shape, 40 kD). In this study, we assessed the efficacy and safety of the Chinese-made peg-IFN α-2b in re-treating patients with HbsAg recurrence after stopping peg-IFN α-based regimens, which we hope will guide the clinical treatment of patients with HBV seroreversion.

## 2. Materials and Methods

### 2.1. Patients

This two-center, prospective observational study was conducted at the Second Affiliated Hospital of Xi’an Jiaotong University and the Ankang Central Hospital between October 2018 and July 2022, and was approved by the Ethics Committee of our Institution (Ethics approval number: 2018-2045; registration number: ChiCTR1900027154). An informed consent form was signed by each patient before their enrolment in this study. Eligible patients for this study were those who had been clinically cured by peg-IFN α-based regimens but experienced HBV relapse after discontinuation of treatment, and were willing to receive peg-IFN α-2b re-treatment. Relapse was defined as the recurrence of either HBsAg or HBV DNA or both at least 2 times at 4–8 weeks intervals following treatment cessation. Patients were excluded from this study if they were co-infected by other hepatotropic viruses or human immunodeficiency virus, or had other forms of chronic liver diseases (decompensated liver cirrhosis, liver cancer, fatty liver, alcohol liver disease, metabolic liver disease, autoimmune hepatitis, HCC), or had any contraindications to peg-IFN α-2b.

### 2.2. Study Design

All enrolled patients were re-treated with180 μg peg-IFN α-2b (PegBeron^®^, subcutaneous injection, once weekly, Xiamen Tebao Biological Engineering Co., Ltd, Xiamen, China). The therapeutic course was individualized and lasted no more than 48 weeks. Briefly, patients could stop treatment at the time of completing 12–24 weeks of consolidation treatment after HBsAg clearance, or at any time during therapy once they were judged as no-responders to peg-IFN α-2b by the clinician. After stopping the peg-IFN α-2b re-treatment, all patients received a 48-week follow-up. Patients with HBsAg clearance could be intramuscularly injected with hepatitis B recombinant vaccine [1 mL (20 HBsAg), GlaxoSmithKline Biologicals] at a dosage of 60 μg/month for 3 consecutive months, according to their own willingness. The main efficacy endpoint was HBsAg clearance at the end of follow-up. The secondary endpoints included HBsAg seroconversion rate, dynamic changes in HBsAg, anti-HBs, alanine aminotransferase (ALT) and liver stiffness measurement (LSM), and the safety of the treatment.

### 2.3. Laboratory Assessments

To closely monitor the efficacy and adverse effects (AEs) of peg-IFN α-2b, blood routine, biochemical parameters, and quantification of serum HBV DNA, HBV biomarkers (HBsAg, anti-HBs, HBeAg, anti-HBe and HBcAb) were performed at baseline and with a flexible interval of ~4–8 weeks during peg-IFN α-2b re-treatment, and with a flexible interval of ~4–12 weeks during follow-up. HBV biomarkers were quantified by using a commercial reagent kit (Architect assay; Abbott Diagnostics) with the lower limit of quantification of 0.05IU/mL. HBsAg clearance is defined as HBsAg < 0.05 IU/mL. HBsAg seroconversion is defined as HBsAg < 0.05 IU/mL accompanied by HBsAb > 10 mIU/mL. Serum HBV DNA was detected using CobasTaqMan96 real-time quantitative PCR detection reagent (Roche), with the lower limit of detection of 20 IU/mL. An automatic biochemical analyzer (Roche, Basel, Switzerland) was used to assay serum ALT, with the upper limit of normal (ULN) at 50 IU/L for men and 40 IU/L for women. In addition, thyroid-stimulating hormone (TSH) was detected at baseline and every 12 weeks during the treatment by using the Cobas e 601 platform (Roche Diagnostics), and LSM was measured by transient elastography (Fibroscan, EchoSens, Paris, France) at baseline and every 24 weeks during the treatment and follow-up.

### 2.4. Statistical Analyses

SPSS software (SPSS 25.0, Chicago, IL, USA) was used for data analysis. Continuous variables were expressed as mean ± standard deviation (SD), or as median (interquartile range [IQR]) as appropriate. Categorical variables were expressed as counts and percentages. The cumulative rates of HBsAg clearance and anti-HBs seroconversion were estimated by using the Kaplan–Meier method. In order to closely reflect real clinical situations, intention-to-treat (ITT) analysis was used in this study.

## 3. Results

### 3.1. Therapy Outcome

**Figure 1:**
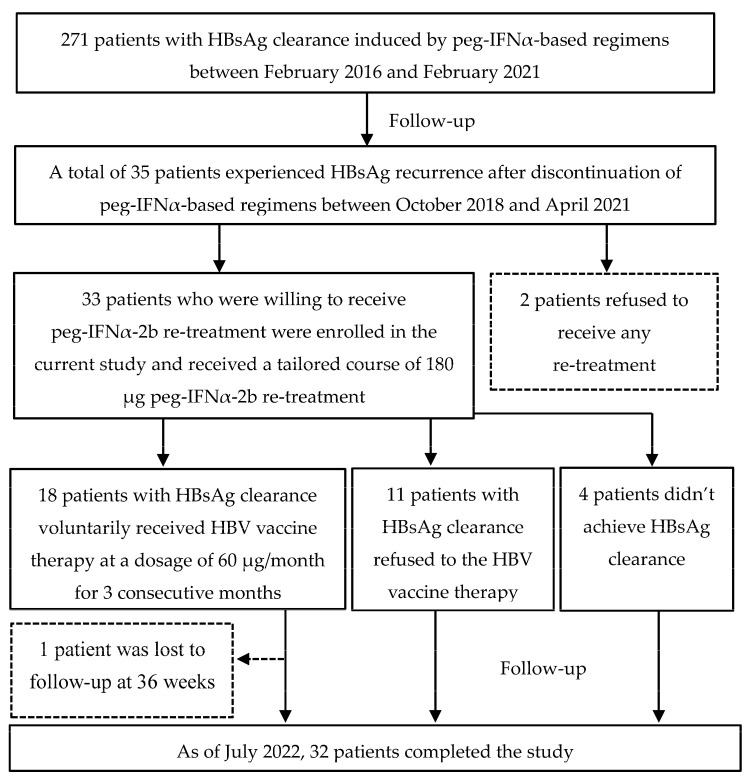
Flow diagram of patients enrolled in this study. (HBsAg, hepatitis B surface antigen; Peg-IFN-α, peginterferon alpha; HBV, hepatitis B virus).

Figure 1 displays the flow chart of participants’ selection, enrollment and follow-up of the current study. Among 271 patients who achieved HBsAg clearance induced by peg-IFN α-based regimens, a total of 35 patients experienced HBsAg recurrence, while no patient experienced HBV DNA recurrence. Finally, 33 patients with HBsAg recurrence were willing to receive peg-IFN α-2b re-treatment and therefore were enrolled in the current study. Approximately 60.6% (20/33) of the patients had a peg-IFN α-2b re-treatment course of ≤24 weeks, and the median re-treatment course was 24 (24–30) weeks (re-treatment course range 20–40 weeks). Of the 33 patients enrolled, 18 patients with HBsAg clearance induced by peg-IFN α-2b re-treatment voluntarily received HBV vaccine therapy at a dosage of 60 μg/month for 3 consecutive months, 11 patients with HBsAg clearance induced by peg-IFN α-2b re-treatment refused HBV vaccine therapy, 4 patients were judged as no-responders to peg-IFN α-2b re-treatment by the clinician and therefore discontinued the peg-IFN α-2b at 24 weeks. During the follow-up period, 1 patient who received HBV vaccine therapy was lost to follow-up at 36 weeks. Therefore, a total of 32 patients completed a tailored course of peg-IFN α-2b re-treatment and scheduled follow-up.

### 3.2. Baseline Characteristics

The baseline characteristics of the 33 patients are summarized in Table 1. Among them, 75.8% (25/33) of the patients had a confirmed family history of chronic HBV infection. The median baseline HBsAg and anti-HBs levels were 1.66 (0.56–2.87) IU/mL (range (0.1–9.1) IU/mL) and 2.58 (1.24–8.51) mIU/mL (range (0.2–74.8) mIU/mL), respectively. All patients were negative for HBeAg and HBV DNA (<20 IU/mL) at the time of enrollment in this study, while 16 (48.5%, 16/33) patients were positive for anti-HBe. Furthermore, all patients had normal baseline clinical characteristics such as total bilirubin (TBIL), ALT, aspartate aminotransferase (AST), white blood cell (WBC), platelet (PLT), alpha-fetoprotein (AFP), TSH and LSM.

### 3.3. Rates of HBsAg Clearance and HBsAg Seroconversion

**Figure 2:**
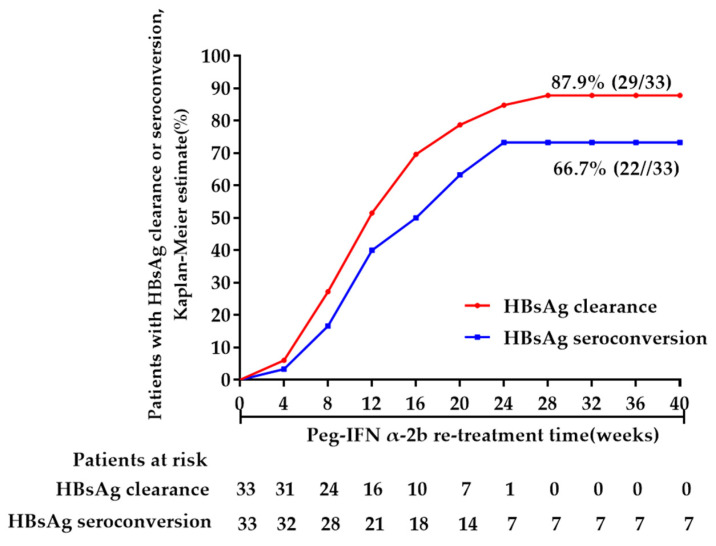
HBsAg clearance and seroconversion during peg-IFNα-2b re-treatment (Week 0 was defined as the time when the patients were enrolled in this study).

Detailed treatment and follow-up information for each patient enrolled in the current study is shown in Table 2. At the end of a median of 24 (24–30) weeks of peg-IFN α-2b re-treatment, 87.9% (29/33) and 66.7% (22/33) of the patients achieved HBsAg clearance and HBsAg seroconversion, respectively (Figure 2). Unfortunately, 2 patients experienced HBsAg recurrence again and 4 patients (including the 2 patients who experienced HBsAg recurrence again) with anti-HBs positive at stopping peg-IFN α-2b re-treatment experienced anti-HBs-negative conversion during the 48 weeks of follow-up. Besides, 1 patient with HBsAg clearance and seroconversion at the end of treatment was lost to follow-up during the follow-up period. None of the 4 patients with HBsAg positive at the end of peg-IFNα-2b re-treatment achieved HBsAg clearance or seroconversion during the follow-up period. Therefore, at the end of follow-up, the HBsAg clearance and HBsAg seroconversion rates decreased to 78.8% (26/33) and 51.5% (17/33), respectively.

### 3.4. Dynamics of HBsAg and Anti-HBs of the 26 Patients Who Maintained HBsAg Clearance during Follow-Up

**Figure 3:**
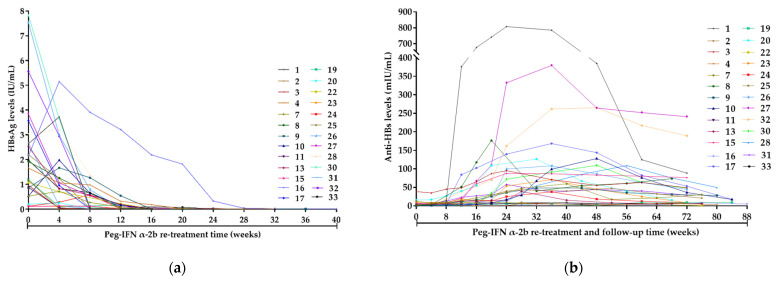
Dynamics of HBsAg and anti-HBs of the 26 patients who maintained HBsAg clearance during follow-up. (**a**) Dynamics of HBsAg of the 26 patients during peg-IFNα-2b re-treatment (These 26 patients remained HBsAg-negative during the follow-up period and are not shown in the figure); (**b**) Dynamics of anti-HBs of the 26 patients during peg-IFNα-2b re-treatment and follow-up. Week 0 was defined as the time when the patients were enrolled in this study. Abbreviations: HBsAg, hepatitis B surface antigen; anti-HBs, hepatitis B surface antibody; Peg-IFN-α, peginterferon alpha.

A total of 26 patients maintained HBsAg clearance during follow-up. Figure 3a,b display the dynamics of HBsAg and anti-HBs of these 26 patients during re-treatment and follow-up. During peg-IFNα-2b re-treatment, the median baseline HBsAg level of these 26 patients was 1.79 (0.78–2.78) IU/mL, which gradually decreased to 0.03 (0.01–0.16) IU/mL at 12 weeks. At 24 weeks, 25 patients achieved HBsAg clearance and 14 completed consolidation therapy and therefore stopped the treatment, while the remaining 11 patients continued consolidation therapy, with the longest therapy course being 36 weeks. Besides, the remaining 1 patient achieved HBsAg clearance at 28 weeks and continued consolidation therapy to 40 weeks, with anti-HBs level of 6.23 mIU/mL at 40 weeks.

The median baseline anti-HBs level of these 26 patients was 2.25 (1.23–6.40) mIU/mL, which gradually increased to 12.31 (3.22–27.28) mIU/mL at 12 weeks and continued to increase to 38.48 (11.67–96.38) mIU/mL at 24 weeks, with 19 patients achieving HBsAg seroconversion and the highest anti-HBs level of 807.83 mIU/mL. The remaining 7 patients did not achieve HBsAg seroconversion during peg-IFNα-2b re-treatment.

During the 48 weeks of follow-up, the median anti-HBs level was 54.23 (8.90–102.05) mIU/mL at week 0, which gradually decreased to 53.97 (7.66–112.77) mIU/mL at week 12, to 46.16 (7.77–83.79) mIU/mL at week 24, to 34.17 (8.03–63.21) mIU/mL at week 36 and to 19.16 (8.21–49.09) mIU/mL at week 48.

### 3.5. Dynamics of HBsAg and Anti-HBs of the Two Patients with HBsAg Recurrence Again

**Figure 4:**
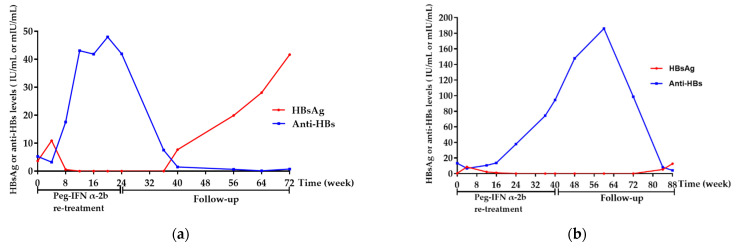
Dynamics of HBsAg and anti-HBs of the two patients with HBsAg recurrence again. (**a**) Dynamics of HBsAg and anti-HBs of the female (No. 5) patient. (**b**) Dynamics of HBsAg and anti-HBs of the male (No. 6) patient. Week 0 was defined as the time when the patients were enrolled in this study. Abbreviations: HBsAg, hepatitis B surface antigen; anti-HBs, hepatitis B surface antibody; Peg-IFN-α, peginterferon alpha.

Figure 4a,b show the dynamic changes in HBsAg and anti-HBs of the 2 patients with HBsAg recurrence again, respectively. Among them, 1 female patient (No. 5) (Figure 4a) with the baseline HBsAg level of 3.65 IU/mL achieved HBsAg clearance again after 12 weeks of peg-IFNα-2b re-treatment and experienced HBsAg recurrence again after 16 weeks of peg-IFNα-2b re-treatment cessation, while another male patient (No. 6) (Figure 4b) with the baseline HBsAg level of 0.47 IU/mL achieved HBsAg clearance again after 24 weeks of peg-IFNα-2b re-treatment and experienced HBsAg recurrence again after 44 weeks of peg-IFNα-2b re-treatment cessation. The anti-HBs levels of these 2 patients rapidly increased with peg-IFNα-2b re-treatment and both patients achieved HBsAg seroconversion. Unfortunately, the anti-HBs gradually decreased after stopping peg-IFNα-2b re-treatment and both patients experienced anti-HBs-negative conversion when HBsAg recurrence. Considering that these 2 patients were clinically cured for the first time by entecavir (ETV) and peg-IFNα-2b combination regimen, ETV was therefore administered to them again to prevent the relapse of HBV DNA.

### 3.6. Dynamics of HbsAg and Anti-HBs of the Four Patients without HbsAg Clearance

**Figure 5:**
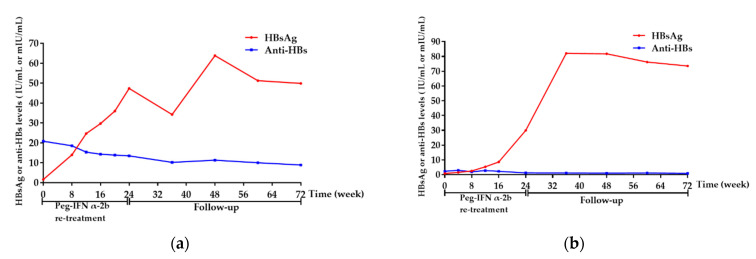

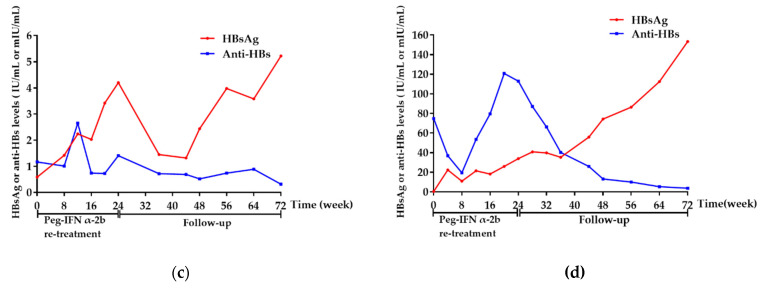
Dynamics of HBsAg and anti-HBs of the 4 patients without HBsAg clearance. (**a**) Dynamics of HBsAg and anti-HBs of the No. 12 patient. (**b**) Dynamics of HBsAg and anti-HBs of the No. 14 patient. (**c**) Dynamics of HBsAg and anti-HBs of the No. 18 patient. (**d**) Dynamics of HBsAg and anti-HBs of the No. 21 patient. Week 0 was defined as the time when the patients were enrolled in this study. Abbreviations: HBsAg, hepatitis B surface antigen; anti-HBs, hepatitis B surface antibody; Peg-IFN-α, peginterferon alpha.

The HBsAg of the remaining 4 patients (No. 12, 14, 18, 21) without HBsAg clearance gradually increased with a 24-week of peg-IFN α-2b re-treatment, as shown in Figure 5a–d, respectively. Considering the poor response to peg-IFNα-2b, treatment was therefore stopped at 24 weeks, according to the clinician’s professional judgment. Among the 4 patients, 3 were clinically cured for the first time by NAs sequential combination with peg-IFNα-2b regimen; therefore, tenofovir disoproxil fumarate (TDF) was administered to them to prevent the relapse of HBV DNA. Another patient refused to receive any further treatment and was only followed-up regularly. Overall, the levels of HbsAg and anti-HBs of these 4 patients showed an increasing and decreasing trend during re-treatment and follow-up, respectively.

### 3.7. Dynamics of HBsAg and Anti-HBs of the One Patient Lost to Follow-Up

One patient, with baseline HBsAg of 0.12 IU/mL and anti-HBs of 9.38 mIU/mL, achieved HBsAg clearance and seroconversion after 12 weeks of peg-IFN α-2b re-treatment, and continued consolidation therapy for 12 weeks. Unfortunately, this patient was lost to follow-up after 36 weeks of follow-up, with HBsAg and anti-HBs of 0.01 IU/mL and 25.12 mIU/mL, respectively.

### 3.8. HBeAg, Anti-HBe and HBV DNA Statuses

The HBeAg and anti-HBe statuses were unchanged in all patients at the time of HBsAg clearance and discontinuation of peg-IFNα-2b re-treatment, as well as at the end of follow-up when compared with baseline. Furthermore, all patients kept the HBV DNA undetectable (<20 IU/mL) throughout the study period.

### 3.9. Dynamics of ALT and LSM

All of the 33 patients had normal baseline ALT with a median level of 21 (9.0–40) IU/L. ALT elevations were observed in 25 (75.8%, 25/33) patients during the peg-IFNα-2b re-treatment, which occurred as early as 4 weeks of peg-IFNα-2b re-treatment. ALT flares (5 × ULN) occurred in 7 patients, with the highest ALT up to 271 IU/L. Gratifyingly, the elevated ALT gradually returned to normal levels after stopping peg-IFNα-2b, with a median level of 20 (9–40) IU/L at the end of follow-up.

The median baseline level of LSM was 6.6 (3.9–11.5) Kpa, which increased to 7.15 (4.8–12.7) Kpa at the cessation of peg-IFNα-2b re-treatment and decreased to 6.2 (4.3–10.7) Kpa at the end of follow-up. No significant statistical change was observed in LSM throughout the study (*p* > 0.05).

### 3.10. HBV Vaccine Injection and Follow-Up

Of the 33 patients, a total of 18 patients received the scheduled HBV vaccine injection immediately after HBsAg clearance. Inspiringly, anti-HBs appeared in 4 patients without anti-HBs at HBsAg clearance, and the levels of anti-HBs significantly increased in 14 patients with anti-HBs positive at HBsAg clearance. Unfortunately, 2 patients with anti-HBs positive at stopping peg-IFN α-2b re-treatment experienced anti-HBs-negative conversion at 24 and 48 weeks of follow-up, respectively. Therefore, about 88.9% (16/18) of the patients with HBsAg clearance benefited from receiving the HBV vaccine therapy. In contrast, only 4 of the 11 patients who cleared HBsAg and refused the vaccine therapy achieved HBsAg seroconversion; the remaining 7 patients maintained the status of HBsAg clearance but anti-HBs negative throughout the study. In addition, 4 patients without HBsAg clearance didn’t receive the vaccine therapy.

### 3.11. Safety

Overall, peg-IFNα-2b was well tolerated except for 2 patients who suffered from sever thyroid function abnormality and needed thyroid medication intervention. Both patients were clinically cured and completed consolidation therapy at 24 weeks, and peg-IFNα-2b was therefore stopped. No patient stopped therapy due to peg-IFNα-related AEs. The most common AEs occurring in this study were pyrexia, headache, myalgia, thrombocytopenia and neutropenia, ALT elevation, fatigue and alopecia, all of which were characteristics of peg-IFN-α therapy (Table 3). Gratifyingly, all of these AEs gradually disappeared after stopping peg-IFNα-2b, and no patient developed cirrhosis or HCC throughout the study. In addition, no HBV vaccine-related AEs occurred in this study.

## 4. Discussion

### 4.1. Short-Term Peg-IFN α-2b Re-Treatment Induced a High Functional Cure Rate in Patients with HBsAg Recurrence

Few studies have been published regarding the re-treatment of patients with HBsAg recurrence after stopping peg-IFN α-based regimens. In this two-center prospective observational study, only patients with HBsAg recurrence after stopping peg-IFN α-based regimens were enrolled and re-treated with a tailored course of Chinese-made peg-IFN α-2b. Our data indicated that a high functional cure rate could be achieved by a short -course of peg-IFN α-2b re-treatment. Furthermore, our study demonstrated the positive role of HBV vaccine in anti-HBs production and HBsAg seroconversion. To the best of our knowledge, this work is the first to focus on the efficacy and safety of peg-IFN α-2b and HBV vaccine in the re-treatment of patients with HBsAg recurrence after discontinuation of peg-IFN α-based regimens.

Peg-IFN-α is the first-line treatment of CHB. It signals through a shared type I IFN heterodimeric receptor complex, comprising two IFN-a receptor subunits (IFNAR1 and IFNAR2) that are present on nearly all nucleated cells [15]. The IFN-IFNAR complex then activates the JAK-STAT pathway, resulting in the expression of dozens of interferon-simulating genes (ISGs), that function as downstream effectors to control viral replication and regulate immune responses [15]. In this study, a high functional cure rate of up to 78.8% could be achieved through a median of 24 (24–30) weeks of peg-IFN α-2b treatment, which was consistent with a recent study reported by Huang et al. [16]. Interestingly, our result was significantly higher than that reported in previous studies conducted on CHB patients treated with peg-IFN α-based regimens for 48 or 72 weeks [6,17], as well as on IHCs treated with peg-IFN α-based regimens for 48 or 96 weeks [8,9]. Patients in the present study had extremely low baseline HBsAg levels (<10 IU/mL) and undetectable HBV DNA may account for the high functional cure rate. However, our result was slightly lower than that reported in a recent study from China that the functional cure rate was up to 86.7% (13/15) in CHB patients who relapsed after cessation of peg-IFN α-based antiviral treatment and were re-treated with peg-IFN-α [18]. Indeed, both studies had a small sample size, which may make the difference in results unable to be explained with confidence. Despite this, both of these studies indicated that a high functional cure rate could be induced by a short-term of peg-IFN-α re-treatment in patients with HBsAg recurrence after stopping peg-IFN α-based regimens.

In addition, intriguingly, all patients enrolled in the current study just experienced HBsAg recurrence and no patient experienced HBV DNA recurrence, which differed from two previously published reports that the cumulative rates of HBV DNA recurrence after stopping peg-IFN-α treatment were 2.33% and 5.04%, respectively [13,14]. Patients in this study were follow-up closely after stopping peg-IFN α-based regimens and were immediately re-treated with peg-IFNα-2b once HBsAg recurrence was confirmed, which may be a possible explanation for the difference in results.

### 4.2. Anti-HBs Reflects the Potent Immune Control of HBV

The production of anti-HBs may reflect more profound immune pressure from the host, resulting in more potent control of HBV [19,20]. Anti-HBs are therefore considered to be an important indicator of functional cure [21]. In this study, a high HBsAg seroconversion rate of up to 51.5% could be achieved through a median of 24 (24–30) weeks of peg-IFN α-2b re-treatment, which was in line with previous studies conducted on CHB patients who were treated with peg-IFN α-based regimens [14]. In addition, the HBsAg seroconversion rate in this study was significantly higher than that induced by NAs in previous studies [11,22]. This rather intriguing result might be partly attributed to the immunoregulatory function of peg-IFN-α [10].

After cessation of peg-IFN α-2b, a decrease in anti-HBs levels was commonly observed and anti-HBs even disappeared in 4 patients, further supporting the notion that anti-HBs levels naturally decrease over time [23]. Furthermore, 2 young patients experienced HBsAg recurrence again accompanied by the disappearance of anti-HBs after cessation of peg-IFN α-2b, indicating that the decline in or disappearance of anti-HBs might reflect the weakened immune control of HBV [14], and have the risk of leading to HBV recurrence.

### 4.3. HBV Vaccine May Contribute to the Production or Elevation of Anti-HBs

Currently, the HBV vaccine therapy has two major application fields: preventing HBV infection and treating patients with chronic HBV infection to enhance the virus-specific immune response and overcome persistent HBV infection [24]. However, few current clinical studies have focused on exploring whether the HBV vaccine therapy can contribute to the production of anti-HBs after HBsAg clearance. Furthermore, except for the application of preventing HBV infection, there is no standard HBV vaccine treatment protocol in clinical application.

In the present study, we made the first attempt to utilize 60 μg/month for 3 consecutive months of HBV vaccine therapy, to enhance the production of anti-HBs in patients with peg-IFN α-induced HBsAg clearance. We found that HBV vaccine therapy can significantly enhance anti-HBs levels, suggesting that the HBV vaccine plays an important role in promoting HBsAg seroconversion or increasing anti-HBs levels after treatment-induced HBsAg clearance [25]. Another important issue to be considered is the safety of vaccine therapy. In the current study, we did not observe any evidence of immune complex mediated disease or deterioration of liver disease as described in some vaccinated wood chucks infected with woodchuck hepatitis virus (WHV), indicating that the HBV vaccine therapy strategy used in this study is relatively safe [24]. These findings will help us to optimize the therapeutic strategies for chronic HBV infection and improve functional cure.

### 4.4. ALT Elevation May Reflect the Immune Activation during Peg-IFN α-2b Re-Treatment

Notably, ALT elevations during peg-IFN α-2b re-treatment occurred in 25 of 33 patients, and 7 patients even experienced ALT flares, which indicates that peg-IFN α-2b may activate the immune response of chronic HBV infected patients, which, in turn, may have contributed to the HBsAg clearance in the current study [25]. The elevated ALT gradually returned to normal levels after stopping peg-IFN α-2b re-treatment.

### 4.5. Study Limitations

Several limitations to this study need to be acknowledged. Firstly, this is a single-arm prospective study with a relatively small sample size (*n* = 33) and lack of a randomized control group. It is worth specifying that large samples of patients with HBsAg recurrence, after being clinically cured by peg-IFN α-based regimens, are difficult to obtain in a relatively short period in the current clinic, due to the fact that peg-IFN α-based regimens are only highly recommended in patients with HBsAg < 1500 IU/mL and HBV DNA undetectable currently [26], and several recent studies have definitively demonstrated that the HBsAg recurrence rate was relatively low (<13%) in patients with peg-IFN α-induced HBsAg clearance during long-term follow-up [14,27]. Furthermore, patients in this study had a strong desire to achieve functional cure again by peg-IFN-α re-treatment. Therefore, it was unacceptable for them to be randomized to the untreated control group. Even so, we believe that enlarging the sample size or setting an un-treated control group will not change the conclusion of this study, because the results of the current study already suggest a significantly higher rate of HBsAg clearance than the untreated spontaneous HBsAg clearance rate, which is well known from many previous studies, that is, only 1%–2.4% per year [28,29]. Secondly, this study had a nonuniform approach of using peg-IFN α-2b. Patients in this study had lower levels of baseline HBsAg (<10 IU/mL) than previous studies. Therefore, in real-world clinical treatment, a tailored course of peg-IFN-α treatment strategy based on HbsAg and anti-HBs response was more reasonable than a fixed 48-week standard course regimen. Thirdly, this study lacked long-term outcome data. The long-term efficacy data are being collected and we will report in the future. Despite these limitations, this work, to the best of our knowledge, is the first prospective cohort study to assess the efficacy and safety of peg-IFN-α re-treatment and HBV vaccine therapy in patients with HBsAg recurrence after cessation of peg-IFN α-based regimens.

### 4.6. Interesting Phenomenon Deserve Further Exploration in the Future

Four patients in this study showed gradually increased HBsAg levels with peg-IFNα-2b re-treatment. It is a puzzling but interesting phenomenon. Further research should be undertaken to understand the possible mechanisms underlying this phenomenon.

## 5. Conclusions

In conclusion, this study demonstrated that a high rate of functional cure could be achieved by a short-course of peg-IFNα-2b re-treatment in patients with HBsAg recurrence after cessation of peg-IFNα-based regimens. HBV vaccine therapy contributes to the production and elevation of anti-HBs. These treatment regimens are relatively safe.

## Figures and Tables

**Table 1:** Baseline demographic and clinical characteristics of the study population.

Characteristics	Patients (*n* = 33)
Male, *n* (%)	18 (54.5)
Age, year	34 (29–40.5)
Had family history of HBV infection, *n* (%)	25 (75.8)
BMI, Kg/m^2^	22.3 (20.1–23.8)
History of treatment, *n* (%)	
NA sequential combination therapy with peg-IFN-α	17 (51.5)
NA de novo combination therapy with peg-IFN-α	3 (9.1)
Peg-IFN-α monotherapy	13 (39.4)
Types of peg-IFN-α in previous study, *n* (%)	
Peg-IFN α-2a	6 (18.2)
Peg-IFN α-2b	27 (81.8)
Follow-up time from discontinuation of treatment	
to HBsAg recurrence, weeks	40 (20–66)
HBsAg, IU/mL	1.66 (0.56–2.87)
Anti-HBs, mIU/mL	2.58 (1.24–-8.51)
HBeAg negative, *n* (%)	33 (100.0)
HBeAb positive, *n* (%)	16 (48.5)
HBV DNA < 20 IU/mL, *n* (%)	33 (100.0)
TBIL, μmol/L	13.2 (10.26–16.57)
ALT, IU/L	21.0 (13.0–28.5)
AST, IU/L	22.0 (18.5–27.0)
WBC, ×10^9^/L	5.36 (4.96–6.51)
PLT, ×10^9^/L	211.0 (165.0–240.0)
AFP, ng/mL	3.55 (2.36–4.79)
TSH, uIU/mL	3.07 (2.71–3.40)
LSM, kPa	6.6 (4.8–8.9)

Data are reported as median [Q1–Q3] and *n* [%]. Abbreviations: BMI, body mass index; NA, nucleos(t)ide analogs; Peg-IFN-α, peginterferon alpha, HBsAg, hepatitis B surface antigen, Anti-HBs, hepatitis B surface antibody; HBeAg, hepatitis B e antigen; HBeAb, hepatitis B e antibody; HBV DNA, hepatitis B virus deoxyribonucleic acid; TBIL, total bilirubin; ALT, alanine aminotransferase; AST, aspartate aminotransferase; WBC, white blood cells; PLT, platelet; AFP, alpha-fetoprotein; TSH, thyroid-stimulating hormone; LSM, liver stiffness measurement.

**Table 2:** Detailed therapeutic and follow-up information for the 33 patients enrolled in this study.

Patient	Gender	Age (Years)	Follow-Up Time (Weeks)	Previous Therapy Strategy	HBsAg Clearance (Weeks)	Consolidation Therapy (Weeks)	Therapy Duration (Weeks)	HBV Vaccine Therapy	HBsAg Seroconversion at Re-Treatment Cession	HBsAg Recurrence	HBsAg Seroconversion at the End of Follow-Up
1	Female	23	72	Peg-IFN	8	16	24	Yes	Yes	No	Yes
2	Male	32	36	ETV+Peg-IFN	4	24	28	Yes	Yes	No	Yes
3	Female	27	16	ETV+Peg-IFN	8	16	24	Yes	Yes	No	Yes
4	Male	52	48	TDF+Peg-IFN	20	12	32	No	No	No	No
5	Female	25	20	ETV+Peg-IFN	12	12	24	Yes	Yes	Yes	No
6	Male	22	40	ETV+Peg-IFN	24	16	40	Yes	Yes	Yes	No
7	Male	35	52	Peg-IFN	16	12	28	Yes	Yes	No	Yes
8	Female	29	44	TDF+Peg-IFN	12	8	20	Yes	Yes	No	Yes
9	Female	29	28	Peg-IFN	16	16	32	Yes	Yes	No	Yes
10	Male	37	8	ETV+Peg-IFN	16	20	36	Yes	Yes	No	Yes
11	Male	30	40	Peg-IFN	8	12	20	No	No	No	No
12 *	Female	40	28	TDF+Peg-IFN	—	—	24	No	No	—	No
13	Female	41	8	ETV+Peg-IFN	16	12	28	Yes	Yes	No	No
14 *	Female	44	12	TDF+Peg-IFN	—	—	24	No	No	—	No
15	Female	29	132	ETV+Peg-IFN	12	12	24	No	Yes	No	Yes
16	Male	40	96	Peg-IFN	28	12	40	No	No	No	No
17	Male	31	32	Peg-IFN	8	28	36	Yes	Yes	No	Yes
18 *	Male	42	16	Peg-IFN	—	—	24	No	No	—	No
19	Female	50	12	Peg-IFN	16	20	36	No	No	No	No
20	Male	34	12	TDF+Peg-IFN	8	12	20	Yes	Yes	No	Yes
21 *	Female	37	76	TDF+Peg-IFN	—	—	24	No	No	—	No
22	Male	25	36	ETV+Peg-IFN	16	12	28	No	No	No	No
23 ^$^	Male	32	20	TDF+Peg-IFN	12	12	24	No	Yes	—	—
24	Female	44	32	Peg-IFN	12	12	24	Yes	Yes	No	No
25	Male	52	60	TDF+Peg-IFN	8	16	24	No	No	No	No
26	Female	25	48	TDF+Peg-IFN	12	12	24	No	Yes	No	Yes
27	Male	30	96	Peg-IFN	12	12	24	Yes	Yes	No	Yes
28	Female	40	60	Peg-IFN	20	12	32	Yes	Yes	No	Yes
29	Male	32	40	ETV+Peg-IFN	12	12	24	Yes	Yes	No	Yes
30	Female	34	86	ETV+Peg-IFN	12	12	24	Yes	Yes	No	Yes
31	Male	44	72	Peg-IFN	8	16	24	No	Yes	No	Yes
32	Male	40	86	ETV+Peg-IFN	8	16	24	Yes	Yes	No	Yes
33	Male	32	28	Peg-IFN	16	12	28	No	No	No	No

12, 14, 18, 21 *: 4 patients without HBsAg clearance during peg-IFNα-2b re-treatment. 23 ^$^: Lost to follow-up during follow-up. Abbreviations: Peg-IFN, peginterferon; TDF, tenofovir disoproxil fumarate; ETV, entecavir.

**Table 3:** Safety of the study population, *n* (%).

AEs	Patients (*n* = 33)
Neutropenia	30 (90.9)
Thrombocytopenia	28 (84.8)
ALT elevation	25 (75.8)
Fever	23 (69.7)
Headache	14 (42.4)
Myalgia	11 (33.3)
Fatigue	10 (30.3)
Anorexia	7 (21.2)
ALT flares	7 (21.2)
Thyroid dysfunction	6 (18.2)
Weight loss	6 (18.2)
Alopecia	4 (12.1)
TBIL elevation	0 (0)
Liver cirrhosis	0 (0)
HCC	0 (0)

Abbreviations: AEs, adverse events; TBIL, total bilirubin; ALT, alanine aminotransferase; HCC: hepatocellular carcinoma.

## Data Availability

Data supporting the findings of this study are not publicly available due to privacy or ethical restrictions and are available upon request to the corresponding author.
